# CARE—Pediatric Colon Adenocarcinoma: A Case Report and Literature Review Comparing Differences in Clinical Features Between Children and Adult Patients

**DOI:** 10.1097/MD.0000000000000503

**Published:** 2015-02-13

**Authors:** King-Jun Koh, Lung-Huang Lin, Shih-Hung Huang, Jia-Uei Wong

**Affiliations:** From the Department of Pediatrics, Sijhih Cathay General Hospital, New Taipei, Taiwan (KJK); Department of Pediatrics (LHL); School of Medicine, Fu Jen Catholic University, New Taipei City, Taiwan (LHL, JUW); Department of Pathology (SHH); and Department of Surgery, Cathay General Hospital, Taipei, Taiwan (JUW).

## Abstract

Colon carcinoma is a rare disease in the pediatric population. Here is a report on a 17-year-old male adolescent with colon adenocarcinoma who presented with recurrent epigastric colic pain for 1 month. Diagnostic laparoscopic surgery revealed a 3.2 × 3 cm tumor at the ascending colon, with serosal involvement and peritoneal metastasis. Clinical differences of colorectal carcinoma among children and adults are reviewed and summarized.

## INTRODUCTION

Colorectal carcinoma (CRC) in children, although rarely discovered, comprises approximately 1% of pediatric neoplasms. It is also the most common primary gastrointestinal malignancy in children.^1^ However, due to the low awareness of the disease, diagnosis is usually delayed until the disease is in the advanced stage, causing prognosis to be extremely poor compared with that of adults.^2^ The presenting symptoms are nonspecific and can mimic those of many benign gastrointestinal conditions in children. However, family history with certain well-documented genetic mutations or inflammatory bowel disease should raise suspicion to the disease.^3^

Here is a report that aims to clarify the clinical features and differences between pediatric and adult colon carcinomas. A clearer insight into the disease may help clinicians arrive at an earlier diagnosis for children and adolescents, and consequently, improve overall survival outcome.

## PRESENTING CONCERNS

A 17-year-old male adolescent suffered from recurrent abdominal colic pain for 1 month. The pain was aggravated by walking and partially relieved by bed rest. No referred pain was noted, but associated symptoms were nausea and mildly loose stool. He denied history of tarry or bloody stool. His father had esophageal cancer.

## CLINICAL FINDINGS

**FIGURE 1:**
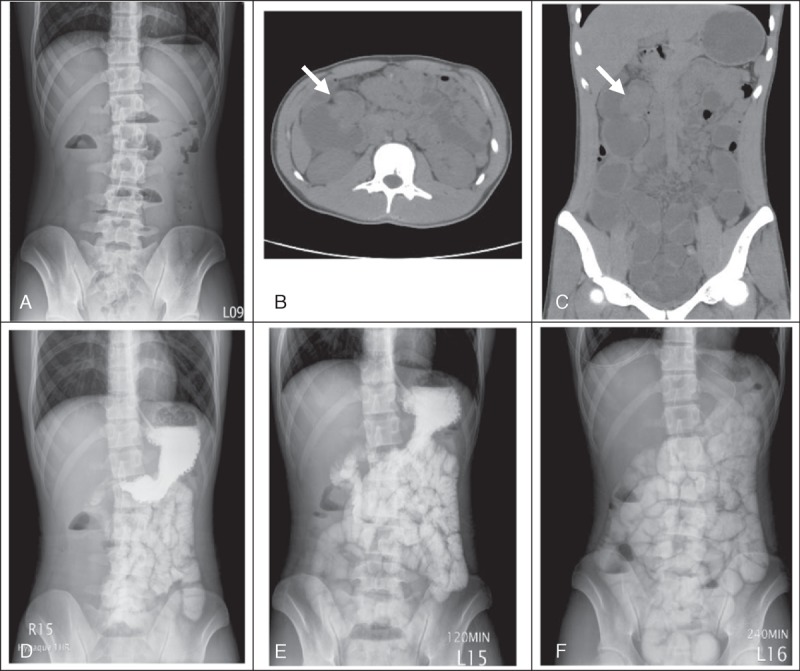
Imaging studies of the patient. (A) Plain abdominal x-ray at initial presentation. (B–C) Horizontal and coronal views of the tumor identified, retrospectively, which was initially masked by the overdistended small intestines. (D–F) Upper gastrointestinal and small bowel series on the first, second, and fourth hour after contrast ingestion, respectively.

Progressive epigastric cramping pain was noted again on the day of admission. There was no fever, vomiting, or diarrhea. Physical examination revealed a distended abdomen with epigastric tenderness. No palpable mass or peritoneal sign was noted. Plain abdomen x-ray showed dilated bowel loops with multiple air-fluid levels (Figure 1A). Intravenous metoclopramide, bisacodyl suppository, and glycerin enema were given but without relief of symptoms.

## DIAGNOSTIC FOCUS AND ASSESSMENT

Abdominal computed tomography (CT) revealed no specific pathologic lesion (Figure 1B–C). Paralytic ileus was the impression. However, there was no clinical improvement even under nil per os and nasogastric decompression. Upper gastrointestinal and small bowel series with water soluble contrast study showed retained contrast media in the small intestine (Figures 1D–F). Mechanical intestinal obstruction was highly suspected.

## THERAPEUTIC FOCUS AND ASSESSMENT

**FIGURE 2:**
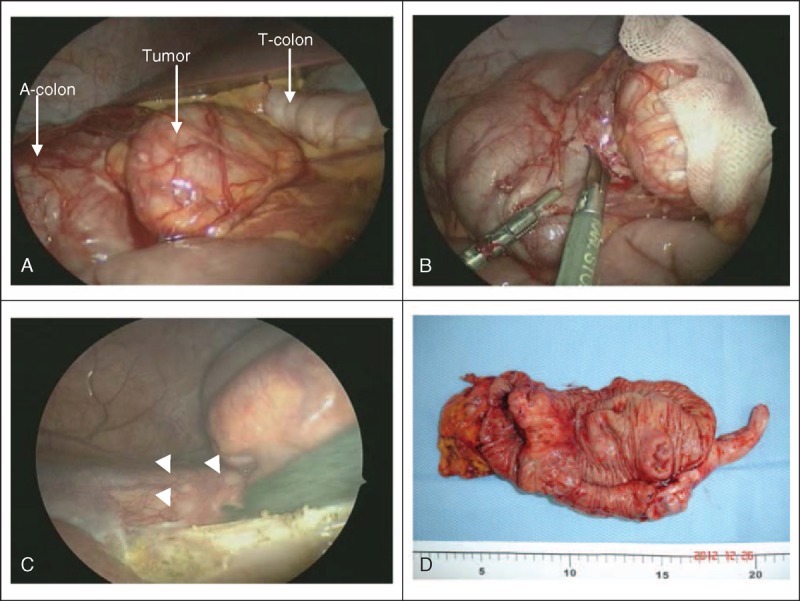
Laparoscopic findings during the surgery. (A) Anatomic location of the tumor in the ascending colon (*A-colon*), adjacent to the transverse colon (*T-colon*). (B) Laparoscopic incision biopsy of the tumor. (C) Carcinomatosis peritonei (*arrow heads*). (D) Macroscopic view of the excised carcinomatous colon tumor with free margins.

**FIGURE 3:**
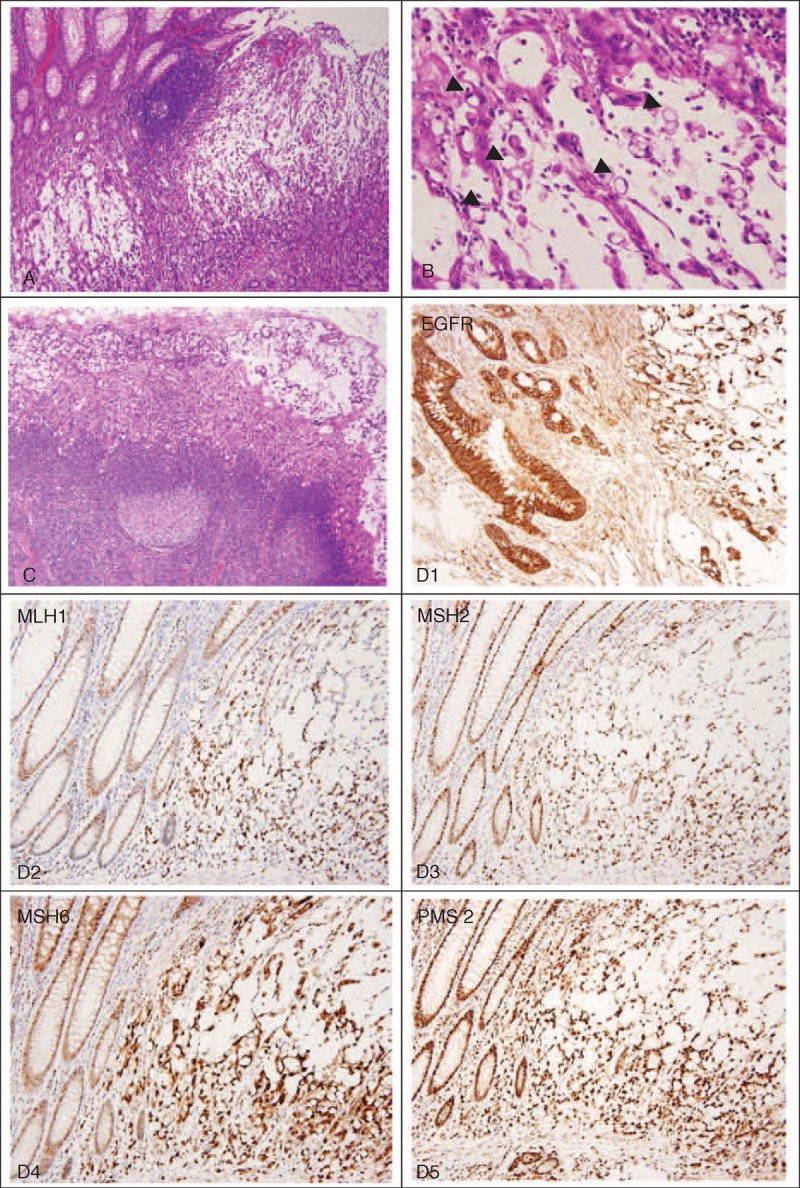
Histologic features of the tumor. (A) Poorly differentiated mucinous adenocarcinoma (hematoxylin and eosin, ×40). (B) Presence of signet-ring cells proportion >10% (arrow heads) (hematoxylin and eosin, ×100). (C) Lymph node metastasis (six of 24 excised lymph nodes). (D1–D5) Immunohistochemical stains are positive for epidermal growth factor receptor (EGFR), and DNA mismatched repair genes *MLH1, MSH2, MSH6,* and *PMS2* have no defects.

Thus, diagnostic laparoscopy surgery was suggested. An annular colon tumor, about 3.2 × 3 cm in size, with serosa involvement, was found in the ascending colon (Figure 2A). Frozen biopsy revealed adenocarcinoma. Right hemicolectomy was performed. Several small peritoneal seeding nodules up to 2 mm were also noted (Figure 2C). The final pathology results showed high-grade, poorly differentiated mucinous adenocarcinoma (Figure 3A) with the presence of signet-ring cells (Figure 3B). Lymph node (6/24) (Figure 3C) and peritoneal metastasis were also confirmed. Final staging was pT4aN2aM1b.

Immunohistochemical staining revealed overexpression of epidermal growth factor receptor (EGFR), which plays an important role in colon cancer oncogenesis and represents an important target for cancer therapy.^4^ However, no defects were found for the deoxyribonucleic acid (DNA) mismatch repair system, that is, the MLH1 (human mutL homolog 1), MSH2 (human mutS homolog 2), MSH6 (human mutS homolog 6), and PMS2 (human postmeiotic segregation 2) proteins (Figure 3D1–D5).

## FOLLOW-UP AND OUTCOMES

Further lung and abdominal CT survey showed no distant metastasis. The patient was then transferred to a pediatric oncology center for chemotherapy with the regimens of bevacizumab and irinotecan. He completed the chemotherapy protocol smoothly without any major complications. After 1 year of follow-up, no tumor recurrence was noted.

## DISCUSSION

CRC is rare in children, with annual incidence of about one in 10 million adolescents younger than 20 years old.^5^ The reported peak age is 15 years old, while the youngest reported patient is a 9-month-old female infant.^6^ Despite its rarity, CRC is the most common primary solid malignancy of the gastrointestinal tract among children.^6^

In contrast to the pediatric population, adult colon carcinoma is the third most common cancer in men and the second in women.^7^ The incidence rate is around 43.7 per 100 000 men and women per year, with peak age of 65 years old.^8^

Abdominal pain and vomiting are the most common symptoms in pediatric CRC patients.^1^ Other reported symptoms include altered bowel habit, weight loss, and anemia.^2^ However, these symptoms are nonspecific in children, which may mimic a lot of common functional gastrointestinal disorders.^9^ The duration of symptoms before diagnosis is usually lengthy, ranging from 2 to 6 months, with median of 3 months.^2^ For elderly patients, symptoms and signs including fecal occult blood test positive (77%), rectal bleeding (58%), anemia (57%), and abdominal pain(52%) were most commonly recorded. The median duration for the above symptoms to diagnosis was 5 weeks (interquartile range 1–20 weeks) (Overall median duration for any symptoms was 14 weeks [interquartile range 5–43 weeks].).^10^ Furthermore, as high as 30% of the adult patients have advantage of early detection by screening.^11^ Other clinical features of pediatric CRC reported in previous literature are summarized in Table 1.

**TABLE 1:**
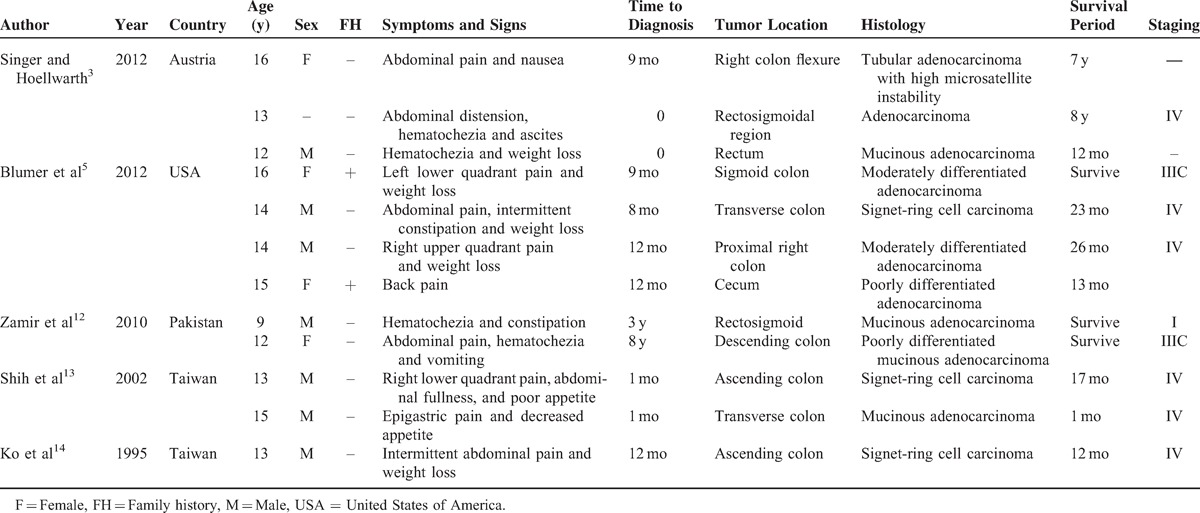
Clinical Characteristics of Pediatric Colorectal Carcinoma in Previous Reported Case Series

The disease usually occurs sporadically.^5^ Nonetheless, 10% to 30% of reported childhood CRCs have predisposing factors,^1^ which include familial adenomatous polyposis (FAP), ulcerative colitis, Crohn disease, and Peutz-Jegher syndrome.^3^ High microsatellite instability (MSI) is one of the most important genetic mutations in childhood CRC^3^ and is caused by a DNA mismatch repair system. It is the hallmark of the early-onset CRC, Lynch syndrome, which is also known as hereditary nonpolyposis colorectal cancer syndrome (HNPCC). Some well-known mutations in DNA mismatch repair genes are *MLH1*, *MSH2*, *MSH6*, and *PMS2*.^15^ On the other hand, mutational inactivation of the *APC (adenomatous polyposis coli)* gene responsible for the FAP is also reported to be higher in adolescents.^9^

The vast majority of CRCs are adenocarcinoma. However, the proportion of poorly differentiated to undifferentiated, mucinous type, signet-ring cell–containing carcinomas is higher in younger patients than in adults. These tumors also behave more aggressively; not only have they a poorer response to chemotherapy, but also associated with extensive intramural spread and peritoneal carcinomatosis.^1^ Moreover, statistics also show a higher incidence of unresectable, residual disease and a higher metastasis rate in childhood CRC.^2^ Other clinical characteristics and differences between childhood and adult CRC are listed in Table 2.

**TABLE 2:**
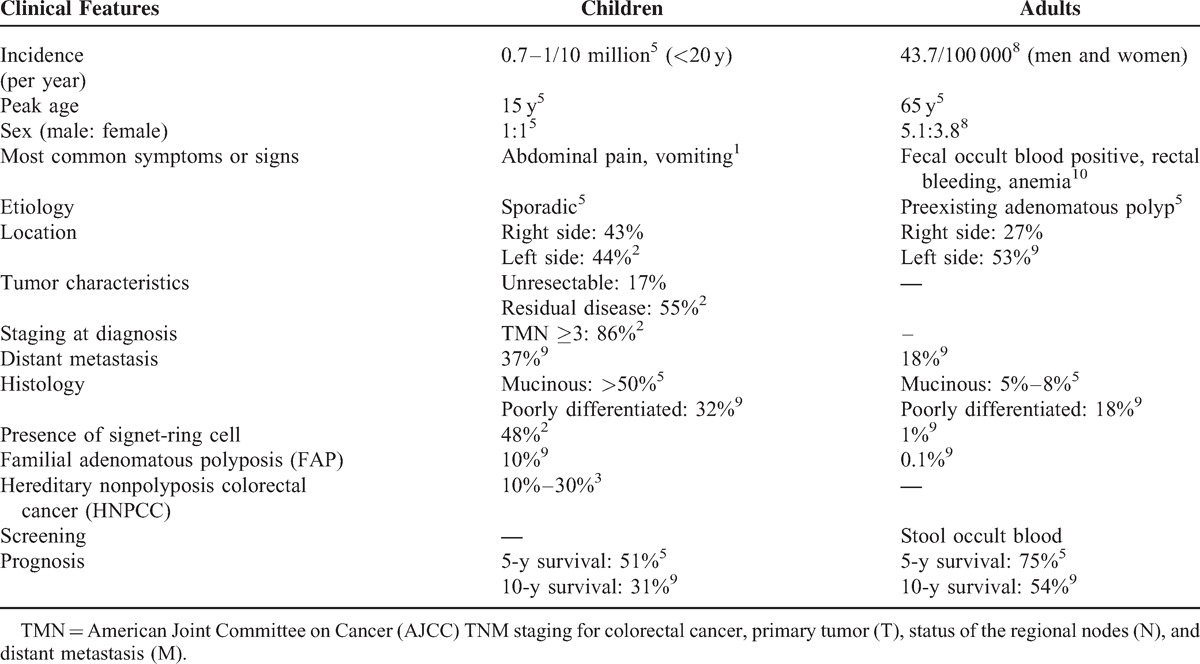
Comparison of Childhood and Adult Colon Carcinoma

Surgery is the only curative modality for localized CRCs. However, for patients with cancer staging ≥III, adjuvant chemotherapy is important to eradicate micrometastases, thereby reducing disease recurrence and increasing the cure rate. Oxaliplatin and 5-fluorouracil–based antineoplastic agents were among the commonly used chemotherapy combinations. In the modern era, monoclonal antibody target therapy such as bevacizumab also showed promising results for metastatic colorectal cancer.

The overall survival in many series reports depends on the complete resection of the tumor and the radical resection of sentinel lymph nodes.^1^ Metastasis occurred in about 18% of newly diagnosed CRCs in adults. However, it might be as high as 37% in young patients even during initial presentation.^9^ The most common distant metastatic sites are liver, lung, lymph nodes, and peritoneum. Predictors of poor outcome aside from disease stage are incomplete resection, mucinous histology, proportion of signet-ring cells >10%, and absence of an in situ component.^2,9^ With the advances in diagnosis and treatments, overall 5-year survival is around 75% in adult patients but only around 51% in pediatric population (Table 3).^5^

**TABLE 3:**
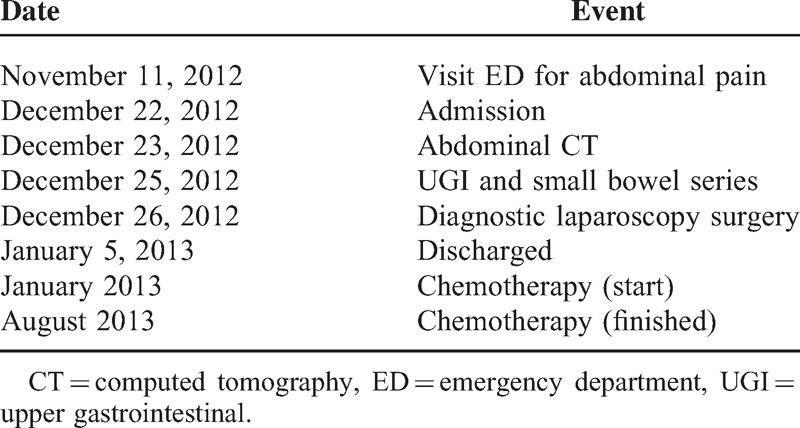
Timeline

## CONCLUSIONS

Awareness and early intervention remain the main challenges in the early diagnosis and prognosis of CRC. Pediatricians and surgeons should have a high index of suspicion in cases of intestinal obstruction without hernia or previous abdominal surgery. In cases of early-onset CRC, testing for high MSI for defects in DNA mismatch repair genes may be helpful for genetic surveillance and prognostic evaluation.
